# Balance is in the Moment

**DOI:** 10.3389/fped.2015.00087

**Published:** 2015-10-13

**Authors:** Michele Y. F. Kong

**Affiliations:** ^1^Department of Pediatrics, University of Alabama at Birmingham, Birmingham, AL, USA

**Keywords:** work-life balance, work-life conflict, working mother, pediatrics, burnout

We do not remember days, we remember moments

Cesare Pavese

Physician burnout is a real phenomenon that impacts us all, even from the early days of our training in medical school, residency, and fellowship (1). In a recent study by Goldhagen et al., the investigators reported an alarmingly high rate of burnout (approximately 80%) among resident physicians (2). As a mother of two young boys, a pediatric intensivist, and a physician scientist, I am acutely aware of the need for work-life balance. How do we strike this mythical balance, when each aspect of our lives, in their respective roles demands our fullest attention? When I am on service in the Pediatric Intensive Care Unit (PICU), my time and energy is entirely focused on my patients. When I am running a critical experiment in the laboratory, successful execution of the procedures is the priority, regardless of the time of day. When I am with the boys, I embrace my role as their mom, teacher, and friend, not as a physician scientist. Is it possible for us to have it all? Physicians, especially those who work in the front line of medicine are more likely to experience burnout and dissatisfaction with their work-life balance compared to the general population (3). The implications are significant, as those in our field are at increased risk for having depression, suicidal ideation, failed relationships, substance abuse, and addiction (4–7). Just as important, these issues can extend to our professional life, with serious consequences on patient care and the health care system (8–10).

For me, balance is found in the *pause* – at that moment when we stop to reflect and consider life. When I first became an attending, I took care of a child^1^ and family who made a profound impact on me. He was 5 years old, and had a neuro-metabolic condition that affected his mobility and left him mostly blind. He presented to the PICU with an acute illness, and despite all our efforts, it became clear that his outcome was poor. It was early in the morning, and I was sitting with the mother near his bedside. There were a lot of hustle and bustle in his room, but for his mom and me, it was quiet. She was telling me about this little boy’s childhood, his love for life despite all his challenges and limitations. Just a few nights prior, he was caught on video, dancing to his favorite band. We laughed together as we watched it, and reflected on the life that he had. He passed away soon after, in the arms of his parents. As physicians, we are trained to fix the problem – if the blood pressure is too low, give more volume or tirate the inotropes. Respiratory failure? Intubate and secure the airway. Renal failure? Initiate dialysis. All necessary steps and procedures, but in our efforts to treat our patients, sometimes we forget about who the patient is, their life and person. The mother later told me that in reflecting on her son’s life, in having that moment during such an overwhelming time of crisis, it helped her in letting go, and in saying goodbye to her son. For me, it reaffirmed the importance of always treating the patient and not the disease – for us all; it is ultimately about life, even in moments of death. I went home the next morning, and spent the day in the park with the boys. Every now and then, especially during an immensely busy day, I pause to reflect on the life that is given to me, and in that moment, I find balance.

Balance is also fluid and dynamic, not static. It is realizing that at any one moment in time, there is a task (or person) that is the most important, and needs to be on the top of our priority list. To maintain balance, it requires us to be flexible and to be able to shift with our priorities. It also means that we are willing to forgive ourselves and to let go of any self-judgment that may surround our decisions at that point in our lives, and accepting that all things cannot be equal in a given space. The notion that we can have it all, and do it all, without any need for priorities is a fallacy. When I was submitting my National Institutes of Health grant, I spent many waking hours and energy on the submission, especially as the dateline drew closer. This meant less time with family and friends, but it also forced me to ensure that whatever time spent was quality time. The ability to prioritize, and to edit our personal and professional life to matters that are of significance is critical in achieving balance.

In this twenty-first century and the era of technology, we are now a generation of physicians who are uber-connected. We are plugged in 24/7, and have the knowledge of our field at our fingertip. We have apps and notifications that alert us to the newest articles and novel findings within our subspecialty. We are connected with our friends and colleagues on social media, and have instant access to our networks online. In such an era of constant connection, it is easy to be caught up in the busyness of life. We learn the value of corporate citizenship, and in our eagerness to be team players, we take on tasks and projects for the sake of the tasks, and nothing more. To have a balance life, perhaps it requires that we get unplugged once in a while. There is extreme value in quiet thinking and solitude. As a distance runner, I value the time spent on trails, before the crack of dawn, with just my thoughts and me. Such a time of solitude often allows me the opportunity to reflect on my life, gain insights, and re-energizes me for the commitment and energy that is necessary to be a successful working mother and physician scientist. Protection of our private time, even if it is a small part of our day is critical to prevent burnout in an already highly charged and stressful career.

Physicians have been reported to work a median of 10 h more per week than the general population, with 37.9% working more than 60 h per week (3). It is not surprising then that 40.1% of physicians report that there was insufficient time for personal or family life (3). In a survey of physicians in the United States and their partners, Dyrbye et al. reported that work-life conflict were more common among female physicians, and those who were younger, worked longer hours, and practiced in academic medicine (11). Numerous strategies have been suggested to reduce this conflict include job sharing, having flexible work hours, stress management, resilience training, nurturing wellness strategies. and seeking work equity policies (12–15). In a randomized clinical trial involving physicians in an academic medical center, a facilitated small-group curriculum provided sustained improvement in meaning and work engagement, with reduced sense of depersonalization (16).

From my vantage point as a working mother, balance requires that we have a strong network of support and collaboration. If nothing else changes, we must be able to embrace the idea that asking for help is not a sign of weakness. Hiring a housekeeper, and having ready-made meals delivered to the house on occasions do not make us less of a parent or spouse. Instead, having the support of these home services allow for less exhaustion and more quality time with family and friends. At work, having advanced practice providers, such as nurse practitioners and physician assistants can contribute to work productivity. In the same way, having a scribe for data entry into the research database allows for more time in the laboratory for research planning and execution. It is critical to have collegiality at work, to be surrounded by a group of colleagues whom we can lean on, share our challenges, and celebrate each other’s success. It means having colleagues who serve as sounding boards, and mentors. It also translates to having peers who can cover our clinical service week when there is a sick child at home, or help in the unit even when they are not on service, an action that is reciprocated when necessary.

To achieve balance, we also need to be content. This does not mean settling for second best, and not striving to be the best that we can in our chosen field. However, it does mean that at any stage that we are in, we need to fully appreciate what we have, and not go through the motions in order to obtain the next paper, grant or promotion. After all, what is the worth of status, or a title if we stop doing things that we love and are passionate about, and have no one to share it with. Happiness is in the moment, and not in the future when we achieve a particular goal. We will find balance if our life and work is driven by purpose and the desire to make an impact in this world. It is possible to love both our work and our life, where there is meaning and significance, even in the smallest task. It is to accept that our definition of balance for today is not the same as for tomorrow as it is a shifting equilibrium. Once we have defined that balance for the moment, we accept the implications and choices that have to be made for supporting our decisions, and know that we are living our life to the fullest.

## Author Contributions

The author contributed to the conception, content, and writing of this work.

## Conflict of Interest Statement

The author declares that the research was conducted in the absence of any commercial or financial relationships that could be construed as a potential conflict of interest.

## Footnotes

^1^All identifying information has been removed
